# An EEG Study on Emotional Intelligence and Advertising Message Effectiveness

**DOI:** 10.3390/bs9080088

**Published:** 2019-08-15

**Authors:** Joseph Ciorciari, Jeffrey Pfeifer, John Gountas

**Affiliations:** 1 Department of Psychological Sciences, Centre for Mental Health, Swinburne University of Technology, Hawthorn Victoria 3122, Australia; 2 Centre for Forensic Behavioural Science, Swinburne University of Technology, Hawthorn Victoria 3122, Australia; 3 Business School, University of Notre Dame, Fremantle 6160, Australia

**Keywords:** EEG, emotional intelligence, consumer decision making, advertising

## Abstract

Some electroencephalography (EEG) studies have investigated emotional intelligence (EI), but none have examined the relationships between EI and commercial advertising messages and related consumer behaviors. This study combines brain (EEG) techniques with an EI psychometric to explore the brain responses associated with a range of advertisements. A group of 45 participants (23 females, 22 males) had their EEG recorded while watching a series of advertisements selected from various marketing categories such as community interests, celebrities, food/drink, and social issues. Participants were also categorized as high or low in emotional intelligence (*n* = 34). The EEG data analysis was centered on rating decision-making in order to measure brain responses associated with advertising information processing for both groups. The findings suggest that participants with high and low emotional intelligence (EI) were attentive to different types of advertising messages. The two EI groups demonstrated preferences for “people” or “object,” related advertising information. This suggests that differences in consumer perception and emotions may suggest why certain advertising material or marketing strategies are effective or not.

## 1. Introduction

Many studies have investigated the neural correlates associated with personality and emotions to better understand consumer behavior and decision-making processes (see review by Kennis et al. [1,2]). Some advertising studies have also suggested that personality traits are reflected in complex decision-making [2,3] and that the poor decision-making associated with personality disorders are possibly explained by deficits in specific personality related neural systems [4] and different levels of intelligence [5,6]. As such, it may be argued that understanding one’s emotions and those of others are critical for successful communications and perceptions, which may also be problematic in those with cognitive or emotional intelligence shortcomings [7]. Even though previous studies have examined the neural basis of emotional traits [8]), personality traits [9] and emotional intelligence [10,11], few have investigated the relationships between emotional intelligence and consumers’ decision making in informative and complex emotion stimuli advertising, such as emotions, celebrity, or sex role portrayal [12,13]. Emotional responses have been used to monitor consumer behavior; however, it is not known how much emotional cognition or intelligence impacts on consumer choice [14].

Emotional intelligence (EI) is the cognitive ability to understand and manage emotions [15]. According to Salovey et al. [16,17], there are different aspects or facets of EI including the ability to appraise emotions and adapt emotional expressions according to situational conditions, cognitively synthesize past emotional experiences into coherent patterns for future application, and generate and regulate the expression of appropriate emotions [16]. People with higher emotional intelligence tend to have greater self-understanding [18], manage amicably interpersonal relations and are more effective in communications [19], and may use moral ethics in decision-making [20]. Several brain regions have been proposed to be part of the functional network associated with the emotional cognition. More specifically, researchers have identified a group of brain regions associated with EI. This includes the amygdala (for appraisal of emotions), the prefrontal cortex (PFC) and anterior cingulate cortex (associated with expression of emotions), and the ventromedial prefrontal cortex (for emotion decision making and self-regulation) [21,22,23,24]. EI competencies are lost when localized frontal lobe damage occurs [25]. More specifically, damage to the ventromedial PFC and dorsolateral PFC decreases strategic and perceptual EI functions that are key components of EI. While performing the Wisconsin Card Sort (WCS) test, strong, positive correlations were reported between the PFC and overall EI performance, further supporting the claim that the frontal lobes (neural basis of intelligence [26]) are associated with EI competencies [24,27].

Electroencephalography (EEG) has been used to identify specific markers of intelligence (IQ) during cognitive processing [28,29] and while making decision associated with advertisements [30]. In particular, frontal EEG changes correlated with differences in IQ [29,30]. Based on the neural efficiency hypothesis, the authors suggested that low IQ groups had a “rigid” type of brain connectivity, which is associated with effortful conscious processing. In contrast, high IQ groups had less “rigid” brain connectivity, suggesting a more fluent and effortless unconscious processing. Similar findings were reported in an EI EEG study, which investigated the relationships with emotional human faces [11] and an emotional words study [31]. Freudenthaler et al. [32] found that low and high emotional management abilities (EMA) were correlated with corresponding event-related EEG differences, corroborating the association between EI with emotional valence and cognition.

The aim of the current study is to extend the EI research testing to more complex stimuli found to be important when investigating advertising media messages with normal participants [30,33] and abnormal behaviors/disorders like compulsive buying [34]. Testing the effects of emotional processing, consumer preferences, and decisions about products and social issues are complex. They are an important research question in assessing the effectiveness of advertising messages [13,35]. Previous neuromarketing studies suggest that consumer behavior and decision-making of product and brand choices are associated with activation in the left inferior PFC activity [36,37,38,39]. However, decisions associated with judgement of social situations and brand tend to be associated with activation of the medial PFC [40]. Emotional intelligence may have an impact on decision making and similar brain regions may be involved; however, the specific neural network may differ with the EI competency.

This study can add some valuable data to the research field on emotional intelligence, sensory processing, and their role in processing commercial advertising by consumers. Even though many factors will influence consumer preference—such as brand information, brand attitude, motivation, social context, and emotion [41]—this study particularly investigates the cognitive emotional processing (EI) and decision-making associated with a range of commercial advertisements.

### Hypotheses

We hypothesize that high- and low-EI groups would have different preferences in the types of advertising messages. High EI are more likely to be interested in processing complex “people themes and social interactions”, and the low-EI participants will be more likely to process “physical objects” themes of messages consistent with previous literature [40]. More specifically, we hypothesize that;

**H1.**  The high-EI cohort will be more interested in themes that deal with social, community, and human interactions rather than functional, technical, product related messages.

**H2.**  In addition, the differences in high/low EI would be reflected in variations of neural connectivity patterns in EEG activity, reflecting preferred emotion processing systems.

## 2. Materials and Methods

### 2.1. Participants

Participants were voluntarily recruited through web-based advertising within the university student and staff community and consented to the project. The sample consisted of 23 females and 22 males with an age range of M = 30.8 ± 11.9 years. Participants also had a total of M = 14.8 ± 1.5 years of education. Because of the special requirements for safe EEG recordings, all potential participants self-excluded if there was a history of epilepsy, brain injury or loss of consciousness, history of psychiatric or neurological conditions, or on medication. Even though they gave consent, participants could withdraw from the study at any time during the experimental session. The university’s ethics committee for human experimentation granted ethics (SUHREC 06/16).

### 2.2. Psychometrics

Prior to EEG testing, participants were required to complete a general demographics survey followed by the validated Swinburne University Emotional Intelligence Test (SUEIT), [10,42]. The SUEIT consists of sixty-five questions which the participants answer on a scale between 0–5 (0 = not at all, and 5 = total agreement). The test can quantify the strengths of emotional qualities with statistical consistency associated with emotion recognition (α = 0.91), understanding of emotions (α = 0.89), direct cognition (α = 0.70), management of emotions (α = 0.83), and emotional control (α = 0.77) [10,42]. From these data, individuals were later categorized into two groupings based on high and low scores in emotional intelligence (EI). The EI groups were grouped into low EI (scores 6.5–28; with 8 males, 9 females) and high EI (scores 40–68; with 8 males and 9 females). The EI groups were determined using a Z score analysis protocol as used by Cheshire et al. [43]. The EI groups were separated into low, middle, and high using Z scores; 33.3% was used to divide the groups. Effect size was calculated using IBM SPSS for the high and low EI (a large effect size; Cohen’s d = 3.27 and Hedges’ gˆ =3.32). Even though all subjects participated in the EEG component of the study, only those included in the high- and low-EI groups (*n* = 34) were tested and their results analyzed accordingly [42].

### 2.3. Electroencephalography (EEG) Stimuli

**Figure 1:**
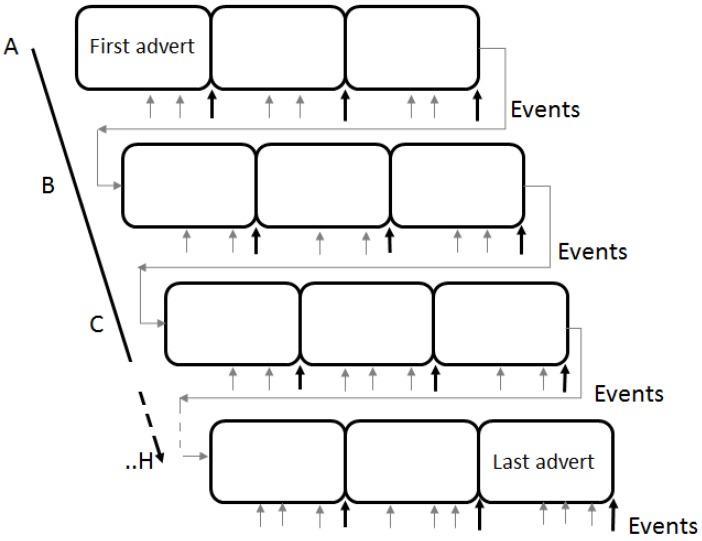
Task design: order of advert stimuli with rating decision-making events. Total 24 adverts per trial (20–30 s each) with continuous breaks (3) and blank pre-stimulus periods of 8 categories adverts (three examples of each in each block A–H were randomized, i.e., 8 × 3). Note for rating decision tasks and for “message” events (grey arrows *n* > 60) there were 72 events. This was repeated 3 times with rest/blank. Participants were asked to note and identify least preferred and most preferred adverts at the end of the advert (black arrows).

Twenty-four commercially aired advertisements were pretested and selected from eight distinct advertising industry themes to be implemented in the experimental protocol. According to Brancaleone et al., these categories are well known from industry and consumers findings [3]. The EEG research paradigm cued randomly all twenty-four adverts, and at the end of viewing each advert, the participants were asked to rate the video in terms of their overall preference. The advertising categories consisted of advertising messages with the following themes: food and drink, pleasure and indulgence items, children and family, social interaction, rural environmental issues, prosocial caring attitudes, new mobile communications, and community affairs. Figure 1 illustrates the design of the automated presentation of the experimental tasks. The adverts from each industry category were used in a random sequence as the task cycled through all categories (3 adverts per block; A–H). At the end of each advert viewing, participants were asked to indicate their overall level preference or overall preference for each advertisement using a Likert scale (0–15; low to high, respectively). High and low preferences were later identified to extract the high (>8) and low (<3) rating preferences for each of the two EI group using a previous approach [43].

### 2.4. EEG Recording

All participants were seated in a comfortable, temperature-controlled room, sitting approximately 50 cm from the display screen, to maintain the same viewing angle. In addition, all stimuli were selected to have similar mean luminance to minimize for luminance changes eliciting visual responses. The EEG were recorded with a Neuroscan EEG system while wearing a 32-electrode cap and watching a series of advertising videos. Firstly, EEG was recorded continuously during two minutes of eyes open and eyes closed rest conditions (baseline) and then during the advert task (24 adverts repeated three times). Electro-oculography (EOG) was also continuously recorded to monitor eye movement to assist with removal of this artifact from the EEG record. All EEG data were recorded, digitized, and processed with Neuroscan SYN-Amp amplifiers and the Neuroscan EEG acquisition software-SCAN version 4.3. The EEG was recorded using a 32-channel standard international 10/20 montage electrode Quickcap. Linked mastoid references and FPz ground leads were used. Typical amplification gain was 100,000 with bandwidth filters set between 0.15 and 200 Hz. Impedance was below 5 kΩ, and EEG was recorded at 1000 samples per second. Frequency bands were defined as delta (0.5–4 Hz), theta (4–8 Hz), alpha (8–14 Hz), and beta (14–25 Hz). Alpha and beta data were used for later calculations of coherence and standard low-resolution electromagnetic tomography (sLORETA) [44], respectively.

### 2.5. EEG Analysis

The EEG method for quantifying functionality across brain sites, referred to as EEG coherence analysis, can help identify and distinguish different types of buying decisions (e.g., compulsive from noncompulsive) according to variations in neural connectivity [34]. Measurements of EEG coherence in executive cognitive processing of visual working memory distinguish processes in decision-making. More specifically, alpha coherence has been a useful measure of brain activity associated with higher functions such as attention, memory, emotion regulation, and engagement [45,46,47,48,49,50]. Specifically, studies have identified specific involvement of prefrontal and fronto-parietal areas in executive functions [45]. While watching television advertising messages, EEG studies have identified different memory and attention processes associated with favorable messages as opposed to unfavorable commercial messages [51]. The EEG coherence analysis technique is employed to map regions associated with information processing. This EEG study also combines brain imaging algorithms (sLORETA) to improve on localization of EEG activity, together with high temporal resolution associated with EEG [44].

EEG was first analyzed offline to remove muscle and ocular artifacts using Brain Vision Analyser’s ICA analysis routine (Brain Products 2004). Neuroscan EDIT (v4.5) was used for subsequent epoching, spectral analyses, and coherence analysis. Previous studies found the EEG to be sensitive to changes in activity and connectivity associated with attention, working memory [45], and intention [52], which would be useful metrics to use for this study. Grand average calculations of alpha and beta spectral data comparing activity during tasks (decision-making) with eyes open EEG (baseline) were performed and prepared for further coherence analyses. EEG data were analyzed to calculate the alpha coherence for each group (high and low EI) during the preferred and nonpreferred advertisement decision-making epochs.

**Figure 2:**
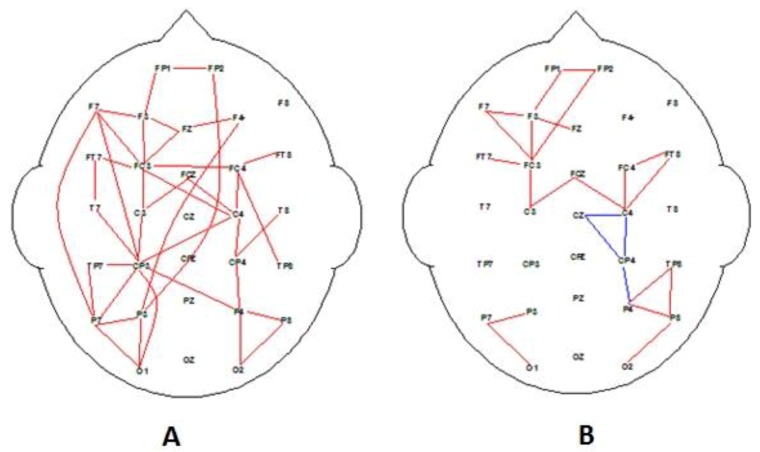
Significant correlation electroencephalography (EEG) coherence maps for group average alpha EEG during decision-making epoch of task for all participants (*n* = 45). (**A**) Significant increase in coherence associated with high-rated stimuli, and (**B**) significant coherence associated with low-rated stimuli adverts. Analysis as per Cook et al. [55]. Note: for all red, (F_1,45_ > 8.9, *p* < 0.01); and for all blue, (F_1,45_ > 7.5, *p* < 0.05).

Alpha power spectra (8–14 Hz) were calculated to measure the degree of desynchronization (decrease in alpha activity) as an index of the cognitive load associated with each decision epoch compared with eyes open rest activity. This phenomenon is consistent with event-related desynchronization (ERD) reported by Pfurtscheller et al. [50]. With ERD, the reductions in alpha amplitude are associated with specific events during the performance of cognitive or motor tasks. Similarly, the EEG epochs associated with the reading and decision phase (at least 3 s) for advert media were averaged and normalized. The three-second epochs are associated with the decision-making epoch, which is consistent with many ERP type studies where a targeted event-related time–frequency analysis is performed [34,53,54]. To further investigate the neural correlates of emotional intelligence, alpha coherence was calculated for each group for preferred and nonpreferred adverts using similar analysis protocol previously reported [34,55]. A montage of 30 bipolar pairs was selected for the coherence analysis, giving even representation of both inter- and intra-hemispheric electrode pairs (see Figure 2). This selection was based on previous cognitive studies described above [34,55]. Alpha coherence analysis was performed offline with Neuroscan Edit 4.5 to extract the EEG alpha coherence phase data for electrode pairs [56]. Coherence analysis was carried out to investigate the correlational strength in regional connectivity between electrode pairs [57]. Coherence analysis reveals the degree of interconnectivity between scalp sites associated with underlying neural processes.

SLORETA analysis is a useful technique that distinguishes relative activity in the different brain regions and estimates the source of activity or deep current densities [44,58]. sLORETA was calculated using Brain Vision Analyser, for the whole viewing period for beta spectral activity (14–25 Hz). Beta activity between active viewing states and rest epochs were compared to determine levels of significance between the epochs using a technique reported by Cook et al. [59].

### 2.6. Statistical Analysis

For each three-second interval (or epoch) of EEG associated with the decision phase of the task and the equivalent EEG during eyes open rest, the measure of alpha coherence for group averaged data was calculated. Statistical analyses relied on the analysis of variance (ANOVA) and using IBM SPSS Statistics (version 24.0), and a 2 (high and low EI) by 30 (electrode pair) ANOVA was conducted for the alpha EEG coherence data. Partial correlations based on the means and standard deviations were calculated for each EEG epoch during a grand mean average of alpha activity and coherence during each decision phase. For the sLORETA calculations, continuous data were analyzed with t-tests, and T-level thresholds were computed corresponding to a threshold of statistical significance (*p* < 0.01) and were noted (2 S.D) [60]. Only these data were used for further sLORETA localization of activity analysis. Parametric analysis was used because the data were normally distributed.

## 3. Results

### 3.1. EEG Findings

**Figure 3:**
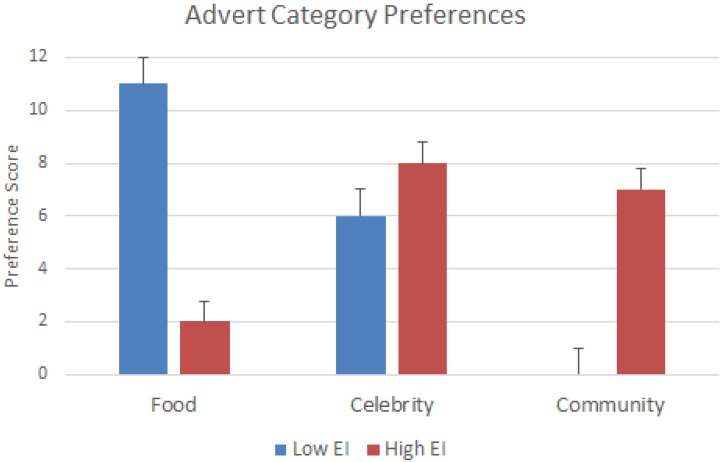
Comparisons of ratings of advert stimuli by high and low emotional intelligence groups. Note *p* < 0.01 for group comparisons. Preferences suggest that the high-EI group had preferences for community- and celebrity-associated adverts, while the low-EI group preferred adverts associated with food products and celebrities.

**Figure 4:**
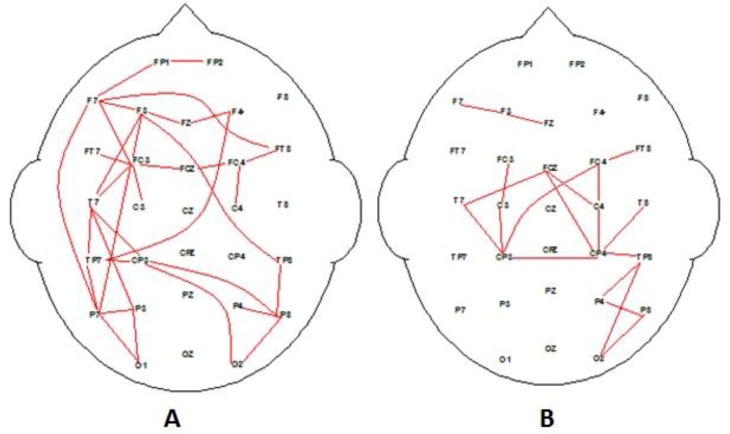
Significant correlation EEG coherence maps for group average alpha EEG during decision-making epoch of task for high-EI participants (increase in coherence). (**A**) Significant coherence associated with high-rated stimuli, and (**B**) significant coherence associated with low-rated stimuli adverts. Analysis as per Cook et al. [55]. Note, *p* < 0.01 (red) and *p* < 0.05 (blue).

**Figure 5:**
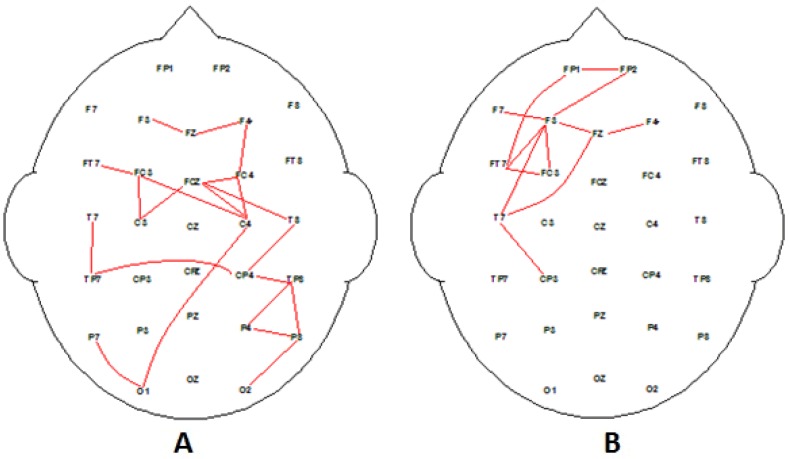
Significant correlation EEG coherence maps for group average alpha EEG during decision-making epoch of task for low-EI participants. (**A**) Significant coherence associated with high-rated stimuli, and (**B**) significant coherence associated with low-rated stimuli adverts. Analysis as per Cook et al. [55]. Note, *p* < 0.01 (red) and *p* < 0.05 (blue).

The coherence data associated with the grand average for decision-making epochs for preferred and nonpreferred adverts are illustrated in Figure 2. Initial analysis reveals distinct coherence differences (increases) associated with the range of different types of advertising messages, whether rated high or low in terms of overall preference: for all red, (F_1,45_ > 8.9, *p* < 0.01); and for all blue, (F_1,45_ > 7.5, *p* < 0.05). However, differences were also seen when groups were divided into high and low EI, illustrated in Figure 3. Figure 4 and Figure 5 support the hypothesis that different networks will be active for the high- and low-EI groups.

One interpretation of the coherence maps illustrated in Figure 2, especially Figure 2A, is that a central executive process involving the fronto-parietal network is active during the decision-making and working memory when evaluating the level of appeal for each advert. Evaluating advertising messages is a complex cognitive task; therefore, more working memory resources are allocated to process the information. Finding activation of the working memory fronto-parietal network is supported by previous research [45]. Our data suggest that the left hemisphere coherence may be interpreted as being associated with the “phonological loop”, while the right hemisphere reflects activation for the visuo-spatial “sketchpad” for visual working memory [38,61]. Both systems were active for the preferred media, but only the visuo-spatial “sketchpad” processed the nonpreferred advertising messages.

### 3.2. Behavioral Findings

In an attempt to better understand advertising communication preferences by high- and low-EI groups, we assumed that high EI would be more interested in social interactions, community issues, ethical buying practices, and people-related themed adverts as supported by previous research [20,62]. In comparison, we assumed that low EI would show higher preference for nonsocial and low for people interaction themes, (e.g., show higher preference for food and industrial functional advertising themes). Figure 3 reports the comparisons of preferred advertising messages by high and low EI. Figure 3 data suggest that high and low emotional intelligence (EI) groups showed clear preferences for types of advertising messages. The high EI indicated much higher preferences for community and social issues advertising themes, but the low EI did indeed prefer adverts with tangible objects (food and drink messages) and less for social- and human-related messages. The most interesting finding is that the high-EI group showed strong preference for “Community and social issues messages”, while the low-EI group did not at all like this theme of message. This finding is supported by Robinson et al. [62] and the role of social messages. Both groups of high and low EI showed interest in the advertising messages that depicted issues of famous and celebrity figures. This probably is due to the strong media presence and high level of popularity and strong influence of celebrities used to promote all kinds of products and causes in all aspects of life in most western societies [63,64].

### 3.3. Combination EEG and Behavioral Investigation

Using the same methodology that produced Figure 2, the EEG data were analyzed for the high-EI group for their highly preferred and nonpreferred advertising messages (see Figure 4). Figure 4 illustrates two very clear connectivity patterns associated with processing information about preferred and nonpreferred media. Also, Figure 4A illustrates connectivity between regions with higher left hemisphere connectivity, with left and right frontal regions when viewing highly liked/preferred advertising messages. Higher EI demonstrates activity and connectivity associated with “person perception”, which involves medial prefrontal cortex, and is consistent with findings from previous studies investigating preference and judgement of social interactions [37] and person judgements [40]. The low-preferred advertising messages are reflected in Figure 4B, which suggest that “non-person” visual processing system is active compared to judgement evaluation processes in the high-preference adverts.

Figure 5 illustrates two very clear connectivity patterns associated with processing information about preferred and nonpreferred advertising messages for the low-EI group. The low-EI group preferred media associated with brands and products (tangible physical objects) producing strong fronto-parietal connectivity during the decision-making process (Figure 5A). The brain activation findings seemed consistent with “Object processing” as reported by other researchers [38]. Generally, products and brands tend to be associated with activation in the left inferior PFC, [37,38,39]. Nonetheless, the coherence maps for the low EI also demonstrates a lower connectivity overall when compared to high EI. In fact, activation of the high EI nonpreferred advertising message brain network in the left frontal–right parietal connectivity (Figure 4B) very closely resembled the low EI preferred messages network activation (Figure 5A). Perhaps low EI are “wired” to process more effortlessly “object” related types of messages rather than “people” related messages.

**Figure 6:**
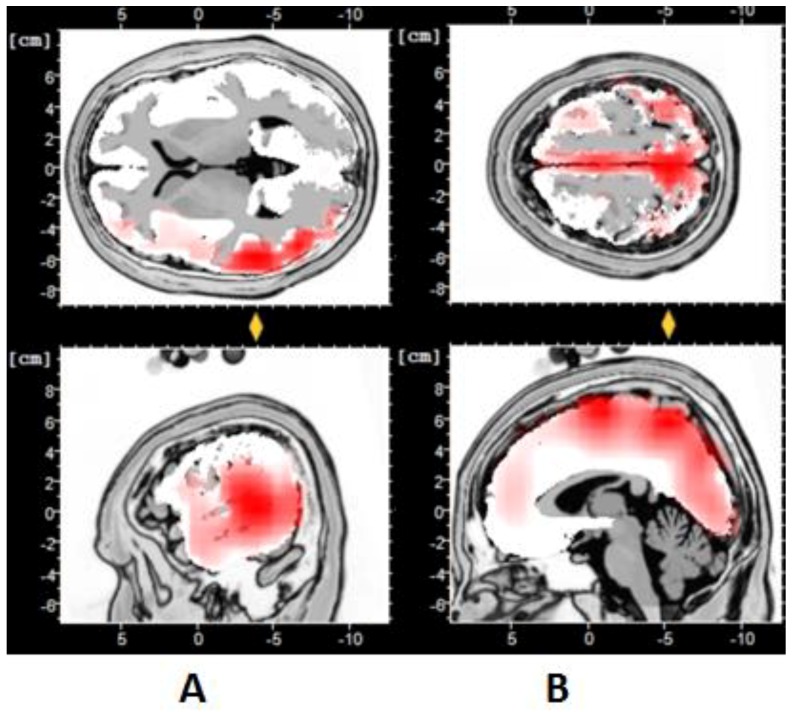
Standard low-resolution electromagnetic tomography (sLORETA) images (beta EEG) for low-EI (**A**) and high-EI (**B**) groups for “messages” epochs of the high-preference adverts. Note top panel (superior view) and bottom panel (sagittal view) for A and B. For A, the significant Regions of Interest (ROI) are Brodmann areas 22 and 37 (language and visual mental imagery processing), while for B, the significant ROI’s are Brodmann areas 6 and 7 (facial association and action observation processing). Note the Student’s *t* test value for this period was *t*(34) = 7.43, *p* = 0.007 for BA6,7 (*p* < 0.01). Note, for maps of significant activity, the unit of current density is μA/mm^2^.

In an attempt to further distinguish high- and low-EI groups, we compared the EEG data that relate to the processing of the different advertisement “theme messages”, for example, comparing the advert message processing for food and drinks versus celebrity and social community issues. sLORETA analysis was performed (see Figure 6) for sources of brain activity for each of the theme message categories (e.g., the advertisements for food, celebrities, and community social issues). The coherence maps of both groups seemed to use different neural resources or brain systems when processing each of the three different “message themes”. The low-EI group (Figure 6A) demonstrated significant activity in Brodmann areas 22 and 37. These regions are associated with language processing and object visual imagery, respectively. This finding suggested that participants engaged primarily in object perception style of mental processing [65]. However, in the high-EI group (see Figure 6B), the main active Brodmann areas were BA6 and BA7. The BA6 region is generally associated with processing symbolic and facial information cues and nonmotor cognitive responses [66]. While BA7 is associated with processing somatosensory, language, and action-related information, which supports the notion that high EI are more interested in the people perception type of information processing [67]. These data support the concept that high EI are primarily “people perception oriented”, while low-EI processing preferences are associated with “object perception”.

## 4. Discussion

One of the main research objectives was to examine decision-making associated with emotional intelligence using an electroencephalographic methodology. The data clearly support the hypothesis that high- and low-EI groups tend to process complex advertising video messages differently, possibly relying on their most efficient neural networks and style of processing [28,29]. Using EEG techniques to quantify connectivity (coherence) and source cognitive processing (sLORETA), the data analysis suggests that distinctly different processing styles are used by high- and low-EI participants during decision-making tasks. The EEG coherence data demonstrated distinct functional connectivity for high- and low-EI groups, which corroborate their associated perceptions, behaviors, and preference ratings of different message themes.

The high-EI group demonstrated clear preferences in specific advertising video messages when contrasted with the low-EI group. The behavioral decision preference data suggest that the high-EI group seemed to demonstrate a “people perception” oriented style of processing, while low EI seemed to demonstrate an “object perception” oriented style of processing, which is consistent with previous ERP research findings [22,68]. Perhaps each high- and low-EI group are “wired” differently to process more or less “object or people” related messages, as it is difficult to distinguish whether information is processed along the dorsal or ventral streams alone [69]. The sLORETA data clearly support this interpretation, because the identified Brodmann areas are related to the processing of object imagery (low EI) and social/people facial imagery (high EI) information processing. Similar differences in cognitive processing styles have been reported in the literature [65,70]. These differences in processing styles (object processing, somatotopic, etc.) have also been shown to be associated with differences in personality and cultural intelligence [70].

The decision-making task for both groups was based on a working memory task methodology [45,61], which demonstrated that fronto-parietal networks were involved suggesting a phonological and visuo-spatial loop or “sketchpads” with a left and right hemisphere difference [38,61]. The EEG data analysis compared the high-rated adverts versus the low-rated adverts for all participants. There is a clear difference for a dominant phonological “sketchpad” with higher left hemisphere fronto-parietal connectivity for high EI and lower fronto-parietal connectivity for low EI (see Figure 2). The two EI cohorts demonstrated evidence of different preferred “sketchpads”. Interestingly, the left frontal connectivity for the low-EI group was dominant for low-rated and nonpreferred media for the high-EI group, demonstrating a clear withdrawal alpha response [49,71] and consistent with previous research supporting the correlation between frontal activity and EI [72].

Perhaps the various connectivity relationships may also reflect personality and personality preferences, as previous literature suggests that certain personality types react differently to imagery, content, and humor [35,72,73,74,75]. The Personality Inventory (NEO-PI) [76] has been used in previous psychophysiology studies of emotional arousal [1,77], but no other study has been done with such complex advertising message stimuli. The use of a multidisciplinary approach (psychometrics and measures of cortical function) can assist in the identification of the neural networks associated with emotions related processes [13], generating insights into individual differences in perception of information processing styles. This study has certainly provided evidence of distinct cognitive and emotional processing styles associated with emotional intelligence [32].

Because of the advantages associated with recording EEG and time locking analysis of key cognitive and advert commercial communication stimuli, this neuroimaging study is a very useful to investigate what the brain is doing during such complex consumer information processing [51,78]. EEG research tools are capable as more expensive fMRI approaches, for producing similar measures of advertising message effectiveness effects and decision-making processes [59,79]. EEG tools are useful to understand how information processing is carried out which can be used to re-construct more effective and engaging advertising messages for prosocial causes like health promotion campaigns [7]. Understanding different cognitive styles is an important factor [80] and can be used effectively in constructing effective commercial messages and appeals for consumer participation with different demographic characteristics [81,82,83]. However careful and ethical usage of EEG research tools is needed to avoid detrimental effects on clinical groups with compulsive buying symptomatology and other psychopathology groups [34].

One of the possible limitations of this study is potential issue of EI and gender. A number of studies have reported clear gender differences in EI [84,85], but this study did not identify similar results even though both EI groups had equal distribution of male and female participants. Perhaps further research with larger cohorts are needed in order to investigate possible gender differences with communications stimuli not equally appealing to both male and female genders [12]. Also, even though data suggests a prosocial bias for the high-EI group, one must question whether self-reporting reflects actual consumer behavior. Personality types should also be investigated as a potential variable in the future [6,21,75].

Another limitation of the study was the form of EEG coherence and sLORETA analysis available to the authors. Even though the same methods have been used by previous studies associated with studying decisions and preferences, more refined methodologies—such as eLORETA (Exact LORETA), nonparametric randomization procedures, and calculating coherence measures in source space (p141) [86]—may have been useful had the means not been normally distributed.

## 5. Conclusions

In conclusion, this study produced potentially useful insights on EI and information processing. High emotional intelligence tends to be associated with “people” focused cognition, producing high frontal–parietal connectivity. In contrast, low EI cognitive processing produced visuo-spatial activation. This study demonstrated the value of using an electroencephalography methodology to explore cognition in cohorts with different emotion processing skills using commercial advertising stimuli. However, the different connectivity patterns do not indicate receptivity and wanting, key factors when addressing preferences for advertising. Nonetheless, this study design may be useful in exploring these factors more closely. There is a possibility that these techniques could be useful in predicting consumer choices. The EEG data demonstrates that there are different decision and perception processes in those who are more emotionally connected. However, it is the authors’ intention that these data generate more hypotheses for future research of the role of emotion regulation and personality to further neuromarketing.
